# Determining the effective timing of an open arthrolysis for post-traumatic elbow stiffness: a retrospective cohort study

**DOI:** 10.1186/s12891-019-2506-3

**Published:** 2019-03-25

**Authors:** Ziyang Sun, Haomin Cui, Jiaming Liang, Juehong Li, Xu Wang, Cunyi Fan

**Affiliations:** 1 Department of Orthopedics, Shanghai Jiao Tong University Affiliated Sixth People’s Hospital, Shanghai, People’s Republic of China; 2 Department of Orthopedics, Shanghai Sixth People’s Hospital East Affiliated to Shanghai University of Medicine & Health Sciences, Shanghai, People’s Republic of China

**Keywords:** Post-traumatic elbow stiffness, Open arthrolysis, Early elbow release, Clinical outcomes, Elbow motion, Elbow function, Heterotopic ossification, Complications

## Abstract

**Background:**

Following trauma, the elbow is the most susceptible to restricted motion among all joints. Open arthrolysis is often performed for post-traumatic elbow stiffness if that stiffness does not improve with non-operative management. However, the optimal timing for performing an open arthrolysis remains controversial. The purpose of this study was to compare the outcome (elbow motion and function) and the rate of complications among patients who had undergone early, median and late release procedures to establish an optimal time interval following the injury, after which, an effective open arthrolysis can be performed.

**Methods:**

In this retrospective cohort study, we included total 133 patients, who had undergone open arthrolysis for post-traumatic elbow stiffness. The subjects were divided into 3 groups, with 31 patients in the early release group (arthrolysis performed at 6–10 months after injury), 78 patients in the median release group (at 11–20 months), and 24 patients in the late release group (at > 20 months). The release procedure in all patients was performed by the same surgeon, using the same technique. The general data, functional performance, and complications, if any, were retrospectively documented for all patients and statistically analysed.

**Results:**

The demographic data and disease characteristics of all patients were comparable at baseline. Postoperatively, no significant differences were found among the three groups with respect to the range of motion (*p* = 0.067), Mayo Elbow Performance Score (*p* = 0.350) and its ratings (*p* = 0.329), visual analog scale score for pain (*p* = 0.227), Dellon classification for ulnar nerve symptoms (*p* = 0.497), and each discrete complication (all *p* values > 0.05).

**Conclusions:**

At the final follow-up, our results showed no significant difference in the postoperative elbow motion capacities, functional scores and the rates of complications among patients who had undergone an early, median, and late release. Therefore, we have recommended that an early arthrolysis would be preferable due to its multiple advantages, and the conventionally observed interval of > 1 year after the injury, could be shortened.

**Level of evidence:**

Level III; Retrospective Cohort Design; Therapeutic Study.

## Background

Post-traumatic elbow stiffness can be diagnosed when range of elbow motion or rotation not meeting patient needs in entertainment, work, and daily life after a related-trauma [1, 2]. The treatment of elbow stiffness is a challenged procedure due to the complicated anatomical manifestation, the involvement of both peri- and intra-articular structures, and a relatively high rate of recurrence and other complications. Surgical arthrolysis can be indicated if the impaired range of motion (ROM) does not improve after an adequate conservative therapy [2–4]. Open elbow arthrolysis has been proved to be effective in restoring functional elbow motion and in achieving a pain-free, stable elbow postoperatively [3, 5]. It is important to evaluate the causes of the contracture before operation by imaging like X-ray and/or computer tomography (CT) scan. Furthermore, the patient must be prepared and motivated to complete an extensive postoperative rehabilitation program.

However, the ideal timing for the performance of an open arthrolysis remains controversial. This is a vital factor in the treatment and rehabilitation of elbow stiffness. Usually, an arthrolysis is performed at ≥6 months after an injury [2–4], during which time bone gradually grows to be mature, and secondary contracture of soft tissue occurs leading to dissatisfactory outcome, or a mature ectopic bone (detected radiologically) forms that can block or tethered the elbow motion [6]. Non-operative management would have little effect at this point. Several studies have reported an early arthrolysis to be both safe and effective [7–9]. Sun et al. reviewed 836 patients across 27 studies with respect to the time interval between injury and surgical elbow release by comparing recurrence rates and the ROM of patients who had undergone early or late release procedures [10]. They divided the patients of all the included studies into 3 groups depending on whether the arthrolysis was performed at 6–10 months, 11–20 months, or > 20 months after the injury. Finally, the mean postoperative Mayo Elbow Performance Score (MEPS) and recurrence rates were found to be similar among the 3 groups. The patients who underwent early arthrolysis (6–10 months after injury) demonstrated the highest gain in ROM and the lowest rate of complications. However, the significance of these differences was not defined. Also, the inhomogeneous data across all the included studies, with respect to parameters such as stiffness severity, surgical techniques, postoperative rehabilitation programs, and the extent of follow-up, would have limited the values of the results.

There are no available guidelines on the optimal time interval after an elbow injury at which point a surgical release can be effectively performed, and few cohort studies relevant to the topic have been published so far. Therefore, the purpose of our study was to compare the elbow motion, function and rate of complications among patients who had undergone early (6–10 months after injury), median (11–20 months after injury) and late (> 20 months after injury) elbow release, to establish the optimal time to perform an open arthrolysis.

## Methods

### Patients and study design

This retrospective cohort study assessed patients with elbow stiffness who presented to our centre between January 2015 and December 2016. Patient medical records were reviewed via an electronic database. The inclusion criteria were: (1) stiffness caused by trauma and (2) patient treated with an open arthrolysis. The exclusion criteria were: (1) < 18 years old at the time of trauma; (2) > 5 year time interval between trauma and arthrolysis; (3) trauma associated with severe burns or central nervous system injuries; (4) trauma associated with non-union or malunion of fractures at the elbow joint; (5) elbow stiffness associated with forearm rotational dysfunction caused by abnormal distal radioulnar joint or forearm interosseous membrane; or (6) history of a previous elbow release procedure. A total of 256 patients underwent surgery for elbow stiffness at our centre during this study period. Of these, 208 met the inclusion criteria, and 59 were discounted as per the exclusion criteria. Of the remaining 149 patients, 16 were excluded because of refusal to participate or as loss to follow-up. The remaining 133 patients were divided into 3 groups with reference to the older study by Sun et al. [10], with 31 patients in the early release group (ER, operated at 6–10 months after injury) [8, 9], 78 in the median release group (MR, operated at 11–20 months), and the remaining 24 in the late release group (LR, operated at > 20 months).

### Clinical evaluation

The patients’ demographic characteristics, history of injury and treatment, elbow function and pain evaluation scores, ulnar nerve symptoms, and heterotopic ossification (HO) status were recorded at baseline. Elbow motion was evaluated using a handheld goniometer. The MEPS and a visual analog scale (VAS) were used for the assessment of elbow function and pain, respectively. The ulnar nerve symptom was evaluated using the Dellon classification [11]. At the final postoperative follow-up in 2018 (with the shortest and the longest follow-up periods at 12 months and 36 months, respectively), all parameters including elbow motion and function, pain, and ulnar nerve symptoms were finally evaluated. The occurrence of postoperative complications such as new onset or exacerbation of nerve symptoms, recurrent HO, elbow instability, wound infection, and surgical pin-related issues were also recorded.

### Surgical technique and postoperative rehabilitation

All patients were operated by the same surgeon (FCY), under general anaesthesia, with the brachial plexus blocked on the operative side using a sterile air tourniquet, applied in the supine position. The surgical incision was selected based on the location of the injury and the presence of any older incision. In general, a combination of lateral-column and posterior-to-medial epicondylar incisions was preferred, unless a patient had undergone prior surgery via a posterior incision. The step-wise surgical process of the arthrolysis was as follows [2]-.

Through a medial approach, the ulnar nerve was routinely identified and released from the ligament of Struthers, proximally, to its entry under the flexor carpi ulnaris, distally. Then, the margins of the triceps tendon were split and reflected off the distal humerus. The posterior band of the medial collateral ligament and posterior capsule were released. Any bony impediments or scar tissue visibly observed within the olecranon fossa were removed, and olecranon fossa plasty or olecranon tip osteotomy was performed if osteophyte was formed around olecranon in order to achieve more improvement in extension. At this point, a pie-crusting technique for triceps tendon release was often utilized to correct a flexion contracture [12]. In the lateral approach, the extensor origins of the brachioradialis and the extensor carpi radialis longus tendons were elevated off the lateral epicondyle. A partial release of the lateral collateral ligament, excision of the hypertrophic anterior capsule, and clearance of radial and coronoid fossae under direct vision were performed. If the patient had presented with a limitation of forearm rotation, the annular ligament and the radio-humeral joint were released, with special attention to the posterior interosseous nerve. A necessary exploration and release of the radial nerve was performed if the patient had severe extension deformity or extensive anterolateral heterotopic bone, or radial nerve symptoms preoperatively.

During the operation, the anterior part of the medial collateral ligament and the ulnar bundle of the lateral collateral ligament were preserved to maintain elbow stability. A contracture release was considered satisfactory if flexion > 130° and extension < 10° were achieved at the elbow. After a complete arthrolysis, the elbow stability was confirmed. Suture anchors or flexor-pronator fascia patches were usually utilized for the collateral ligament repair [13, 14]. Anterior ulnar-nerve transposition was performed subcutaneously in all patients [15, 16]. The arc of elbow motion was again re-examined, and a hinged external fixator (Orthofix, Verona, Italy) was applied [14, 17, 18]. Two drainage tubes were left in place to prevent hematoma, and the wound was closed in layers after local application of vancomycin powder [19]. The elbow ROM was documented in all patients to guide individual rehabilitation.

All patients underwent a postoperative rehabilitation program in three stages. 1) The first stage extended from postoperative day 1 to 6 weeks. We prescribed celecoxib (200 mg orally, twice daily) to prevent HO and for pain control. Patients were instructed to lift the upper limb and perform active muscle contraction. All patients were treated by the same doctor (Wei Wang), one of the member in our “*Elbow Dysfunction Treatment Team”*, and started on an exercise program on postoperative day 1, under supervision. The exercises consisted of circles of passive, assisted, and active elbow flexion and extension, with 30 movements on the first day, increased by a count of 30 per day until 300 movements were achieved; and the forearm rotation exercises were performed twice a day, after a temporary removal of the external fixator, which was re-applied by the same doctor (Wei Wang) after the session. 2) The hinged external fixator was removed at 6 weeks in the outpatient operating room, at which point the second stage of rehabilitation was initiated, which lasted up to 3 months. In this stage, besides flexion and extension exercises, a systematic program for the rehabilitation of forearm rotation was initiated. 3) In the third stage, from 3 months to 1 year, patients were instructed to continue the physical therapy (for minimum 30 min, 3 times a day), and weight-bearing exercises were encouraged, starting with a 1 kg weight, under supervision.

### Statistical analysis

The continuous variables were presented as mean ± standard deviation (range) when they were normally distributed. Alternatively, the median and interquartile range were reported. The qualitative variables were presented as numbers and percentages. An analysis of variance (ANOVA) or the Kruskal-Wallis test was used to compare continuous data. The Kruskal–Wallis, Fisher’s exact, or Pearson’s χ^2^ tests were used to compare qualitative data. The derived *p*-values < 0.05 were considered statistically significant. The statistical analysis was performed using IBM SPSS software (version 22.0; IBM, Armonk, NY, USA).

## Results

**Fig. 1:**
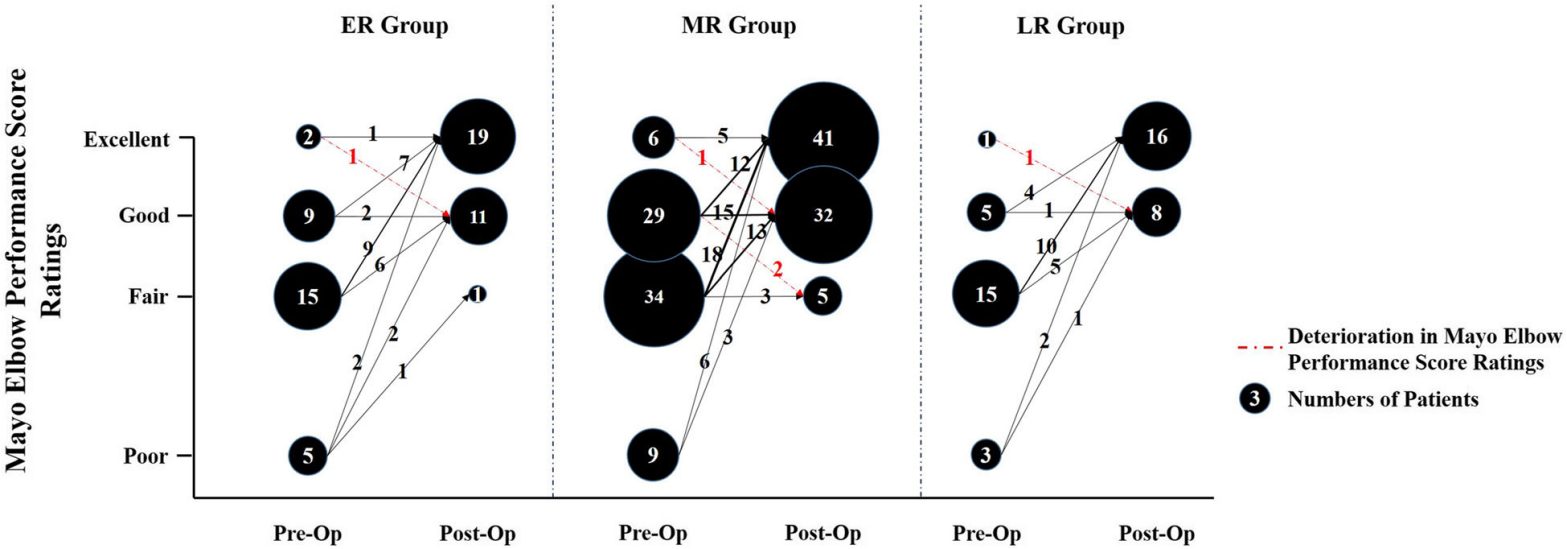
The improvement of preoperative to postoperative Mayo Elbow Performance Score Ratings for elbow function is shown here. The MEPS ratings for almost patients were good-excellent, with a percentage of 97, 94, 100% in ER, MR, LR groups respectively, and 95% in the total cohorts. *ER* early release group, *MR* median release group, *LR* late release group, *Pre-op* pre-operation, *Post-Op* post-operation

**Fig. 2:**
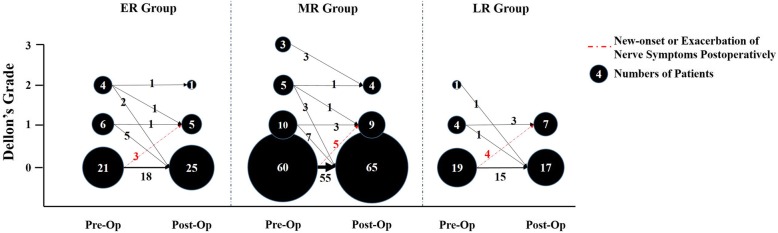
The improvement of preoperative to postoperative Dellon’s Grade for ulnar nerve symptoms is shown here. A total of 26 patients complained of 26 patients complained of ulnar-nerve symptoms at the last follow-up, with 6 patients in ER group, 13 in MR group, and 7 in LR group, respectively. Among these, 12 patients were new-onset or exacerbated nerve symptoms, with 3 in ER group, 5 in MR group, and 4 in LR group. *ER* early release group, *MR* median release group, *LR* late release group, *Pre-op* pre-operation, *Post-Op* post-operation

The demographic data, clinical characteristics, and preoperative data of all patients were comparable at baseline (Tables 1 and 2). During the final follow-up, significant improvements were observed in all evaluated parameters (Tables 2 and 3, Figs. 1 and 2). Of these, the physical examination findings showed no statistically significant differences among the three groups in the evaluation of extension, flexion, and ROM of the operated elbow. ROM of the ER group (122 ± 18) was found to be slightly higher than that of the MR (114 ± 18) and the LR (120 ± 17) groups (Table 3). As for the clinical function assessment, the MEPS scores for almost patients were good-excellent with no significant differences observed between the scores (*p* = 0.350; Table 3) and score ratings (*p* = 0.329; Table 3, Fig. 1), among the three groups. The mean VAS pain scores in ER, MR and LR groups were also comparable, with no significant difference among the three groups (*p* = 0.227; Table 3). Total 26 patients complained of ulnar-nerve symptoms at the final follow-up. Figure 2 shows the distribution of these patients in the ER, MR, and LR groups respectively, recorded as Dellon classification [11]. No significant difference was observed among the three groups (*p* = 0.497; Table 3) in this criterion either.

**Table 1:** Demographics and clinical characteristics of patients

Characteristics	ER	MR	LR	*P* value
No. of patients	31	78	24	
Male, n	22 (71)	49 (63)	16 (67)	.715
Age, years	36 ± 12	37 ± 11	39 ± 11	.587
Body mass index, kg/m2	22.4 ± 3.4	23.4 ± 2.8	23.5 ± 3.1	.244
Timing point^†^, months	9, 7–10	15, 12–17	26, 24–37	<.001^*^
Dominant Limb, n	16 (52)	48 (62)	14 (58)	.637
Initial injury, n				.856
Singular fracture	26 (84)	62 (79)	19 (79)	
Combined fractures	5 (16)	16 (21)	5 (21)	
Treatment history, n				.187
Operative therapy	25 (81)	71 (91)	19 (79)	
Conservative therapy	6 (19)	7 (9)	5 (21)	
Follow-up Time^#^, months	21 ± 6	23 ± 7	22 ± 7	.252

*ER* early release group *MR* median release group, *LR* late release group

^†^Length of time from trauma to arthrolysis

^#^Postoperative period after elbow release (the final postoperative follow-up in 2018, with the shortest and the longest follow-up periods at 12 months and 36 months, respectively)

Categorical variables are presented as number (%)

Continuous variables are presented as mean ± standard deviation, or median, interquartile range

^*^P < .001

**Table 2:** Clinical evaluation of patients: preoperative data

Characteristics	ER	MR	LR	*P* value
No. of patients	31	78	24	
Extension, °	36 ± 17	39 ± 19	42 ± 19	.430
Flexion, °	76 ± 23	80 ± 21	81 ± 23	.602
ROM, °	40 ± 27	41 ± 26	39 ± 30	.957
MEPS, points	66 ± 15	70 ± 12	67 ± 11	.342
Excellent, n	2 (6)	6 (8)	1 (4)	.297
Good, n	9 (29)	29 (37)	5 (21)	
Fair, n	15 (48)	34 (44)	15 (63)	
Poor, n	5 (16)	9 (12)	3 (13)	
Pain VAS, points	1.7 ± 2.0	1.5 ± 1.9	1.6 ± 2.1	.870
Ulnar nerve symptoms (Dellon classification)				.541
None, n	21 (68)	60 (77)	19 (79)	
Grade I, n	6 (19)	10 (13)	4 (17)	
Grade II, n	4 (13)	5 (6)	1 (4)	
Grade III, n	0 (0)	3 (4)	0 (0)	
HO (Hastings and Graham classification)				.849
None, n	8 (26)	17 (22)	6 (25)	
Grade IIA, n	14 (45)	34 (44)	9 (38)	
Grade IIC, n	4 (13)	14 (18)	4 (17)	
Grade III, n	5 (16)	13 (17)	5 (21)	

*ER* early release group, *MR* median release group, *LR* late release group, *ROM* range of motion, *MEPS* Mayo Elbow Performance Score, *VAS* visual analog scale

Categorical variables are presented as number (%)

Continuous variables are presented as mean ± standard deviation

**Table 3:** Clinical evaluation of patients: postoperative data

Characteristics	ER	MR	LR	*P* value
No. of patients	31	78	24	
Extension, °	6 ± 9	11 ± 14	9 ± 10	.205
Flexion, °	129 ± 11	125 ± 9	128 ± 9	.094
ROM, °	122 ± 18	114 ± 18	120 ± 17	.067
MEPS, points	93 ± 9	91 ± 10	94 ± 8	.350
Excellent, n	19 (61)	41 (52)	16 (67)	.329
Good, n	11 (35)	32 (41)	8 (33)	
Fair, n	1 (3)	5 (6)	0 (0)	
Pain VAS, points	0.9 ± 1.2	1.3 ± 1.7	0.8 ± 1.2	.227
Ulnar nerve symptoms (Dellon classification)				.497
None, n	25 (81)	65 (83)	17 (71)	
Grade I, n	5 (16)	9 (11)	7 (29)	
Grade II, n	1 (3)	4 (5)	0 (0)	

*ER* early release group, *MR* median release group, *LR* late release group, *ROM* range of motion, *MEPS* Mayo Elbow Performance Score, *VAS* visual analog scale

The final postoperative follow-up in 2018, with the shortest and the longest follow-up periods at 12 months and 36 months, respectively

Categorical variables are presented as number (%)

Continuous variables are presented as mean ± standard deviation

New-onset or exacerbated nerve symptoms, recurrent HO, elbow instability, wound infection, and pin-related issues were identified as postoperative complications (Table 4). No significant difference with respect to new-onset or exacerbated nerve symptoms was observed among the three groups (*p* = 0.308; Fig. 2). Furthermore, no recurrent HO was found in the ER group as compared to the MR group, which included 3 patients (2 with grade IIA and 1 with grade IIC), and the LR group, which included 2 patients with (grade IIA) HO. However, this finding was not statistically significant (*p* = 0.275). Only 1 patient had moderate elbow instability in the LR group (< 10° of varus-valgus laxity), which was observed to have been spontaneously corrected by the final follow-up. No significant differences were found with respect to both pin-related complications (*p* = 0.249) and wound infections *(p =* 0*.*566) among the three groups, either.

**Table 4:** The distribution of postoperative complications

Characteristics	ER	MR	LR	*P* value
No. of patients	31	78	24	
Nerve complications^a^	3 (10)	5 (6)	4 (17)	.308
Ulnar nerve	3 (10)	5 (6)	4 (17)	
Radial nerve	0 (0)	0 (0)	0 (0)	
Median nerve	0 (0)	0 (0)	0 (0)	
Recurrent heterotopic ossification	0 (0)	3 (4)	2 (8)	.275
Elbow instability	0 (0)	0 (0)	1 (4)	.103
Wound infection	0 (0)	2 (3)	1 (4)	.566
Pin-related complications	3 (10)	2 (3)	2 (8)	.249
Others^b^	1 (3)	3 (4)	1 (4)	.982

*ER* early release group, *MR* median release group, *LR* late release group

^a^new-onset or exacerbation of nerve symptoms postoperatively

^b^including refracture, hematoma, synovitis, tricep avulsion, incomplete wound healing with a sinus, wound dehiscence and intrarticular bleeding

Categorical variables are presented as number (%)

## Discussion

The elbow is more susceptible to motion loss than any other joint after trauma [20, 21]. The aim of treatment for elbow stiffness due to trauma, is to achieve a functional range of movement and a pain-free, stable joint [1–4, 22]. The disorder can be treated nonoperatively or surgically. A failed trial of non-operative therapy is a strong indication for performing a surgical release [2–4]. Depending on the operating surgeon’s expertise level in elbow arthroscopy, status of the ulnar nerve, formation and location of HOs, extent of the contracture, and articular surface damage, arthroscopic techniques may be preferred to relieve simple contractures [23, 24]. Therefore, an open arthrolysis is the most commonly reported, traditional treatment method [2, 25, 26], using which, a mean ROM of 103° (Range: 85°–129°) and a mean gain of 51° (Range: 38°–60°) have been achieved, with a mean complication rate of 23% (Range: 0–59%), as reported in a systematic review of 637 operated patients across 21 different studies [3].

There is a lack of evidence regarding the optimal time interval from the time of injury after which, a surgical release is indicated. Older studies have advocated a longer waiting time of 12–24 months (from injury to operation), in order to ensure the maturation of bone and reduce the recurrence rate. However, these studies have ignored the progressive fibrosis of the surrounding soft tissues, like capsules, collateral ligaments, and muscles, during this waiting period [27]. Muscle atrophy can also appear when range of motion at the elbow is limited over a long period, which would negatively impact the postoperative rehabilitation program, wherein active cycles of flexion-extension and forearm rotation exercises are encouraged and are required to be performed by each patient [28, 29]. Patients with symptoms of ulnar nerve motor dysfunction (with or without disability) will be at a higher risk of aggravation of the nerve injury due to ischemia and compression, caused by a constant dysfunctional extension at the elbow, or following continuous pressure from a surrounding HO [30, 31]. The articular cartilage also deserves special attention, as it could be damaged and destroyed when the elbow is immobilized, or limited to a small ROM, or hinged acting with an abnormal articular surface or structure (such as trochlea) over a long period [32, 33]. Such degradation of the articular cartilage would contribute to secondary joint arthrosis, which in turn, will further aggravate elbow stiffness, pain, and instability. Additionally, a long-term stiffness of the elbow would significantly inconvenience patients in their daily activities and has a great negative impact on the quality of life [34, 35]. Most patients would want to resolve this issue as early as possible.

Sun et al. found in their systematic review of 27 studies with 836 patients, published between 1989 and 2017 that the patients operated earlier (surgical timing: 6–10 months) achieved the highest mean gain in ROM (71°, from 39° preoperatively to 110° at the final follow-up), which was higher than that achieved by the patients operated at 11–20 months (62°) and at > 20 months (58°), respectively. The mean rate of complications was also lower in the group operated earlier (17.0%), as compared to the median (22.7%) and the long (21.4%) interval groups. However, the statistical significance of these differences was not analysed in their study. They recommended an early surgical release, so that the patient could have a shorter rehabilitative period and could return to work earlier [10]. The results of our study are similar. There were no significant differences in postoperative elbow ROM *(p =* 0*.*067), MEPS *(p =* 0*.*350) and its score ratings *(p =* 0*.*329), pain level *(p =* 0*.*227), ulnar nerve symptom *(p =* 0*.*187), and individual complication rates (all *p* values > 0.05), among the three ER, MR and LR groups. This meant that an early arthrolysis would not negatively affect postoperative elbow motion capacity and functional outcome. Also, the risk of postoperative complications would be the same as with a late arthrolysis procedure. In another relevant study, Haglin found that patients who achieved an ROM of at least 100° postoperatively had a significantly shorter mean time interval from initial injury to initial arthrolysis than in those who had failed to achieve a similar ROM (35 vs. 103 weeks, *p* < 0.0005) [25]. Koh also reported that the time from the initial injury to surgical release, with a cut-off value at 19 months, was the only independent factor affecting the final elbow ROM [36]. More importantly, Zheng reported that a long period of elbow stiffness (> 5 years) could negatively influence the functional outcome (MEPS, *p* = 0.016) and increase the risk of complications *(p* = 0.002) after a subsequent arthrolysis.

Traditionally, surgeons waited for at least 1 year after initial trauma to intervene surgically, due to a high risk of recurrence of HO [37]. Recent studies have however, reported good results following an early excision (at < 1 year) with no difference in the HO recurrence rates as compared to a delayed surgery [7, 38]. Chen retrospectively reviewed 164 patients with HO, who underwent open arthrolysis, and divided them into two groups, with the HO excision performed at an average 23.0 months after initial injury (range: 9–204 months) and an early excision group, who underwent the excision procedure at average 6.1 months (range: 3–8 months). They finally found no significant difference between the two groups with respect to postoperative ROM, MEPS and complications, especially for HO recurrence (*p* = 0.942) [7]. Similarly, in our study, no significant difference in HO recurrence (*p* = 0.275) was found among the three groups. The HOs generally mature in approximately 3 to 6 months [39], and based on our findings, we would recommend an early excision at 6 to 10 months after the initial injury. However, it is important to wait until a mature HO, with well-demarcated cortical margins and trabeculations is observed radiologically, and signs of new bone formation such as pain, local tenderness, swelling, and hyperaemia have resolved. A careful management of the HO intraoperatively with an appropriate postoperative rehabilitation regimen (rather than implementing stressful and violent exercise), is essential.

For now, there is a consensus that non-operative treatment should be attempted for at least 6 months, before considering an operative modality [2–4]. Non-operative treatment methods like dynamic or static progressive splinting may be effective, possibly due to the mechanism of soft-tissue relaxation. In patients with a relatively mild contractures, following an elbow injury sustained < 6 months back, there has been a reported improvement of 35–50° in elbow ROM with conservative management [40]. Currently, the utilization of an active or passive stretching of the elbow during exercise is under debate. Several authors have discussed the use of CPM (Continuous Passive Motion) devices in the postoperative phase, to prevent recurrent elbow joint stiffness [22, 41, 42]. Although there are concerns that passive-aggressive elbow exercises may lead to an increased likelihood of heterotopic bone formation, there is little evidence to clarify the relationship between passive motion and HO. We advise that passive range of motion exercises should be performed progressively, and under supervision, with a low load and within tissue tolerance. The patient should be relaxed and should not experience much pain when passive stretching is applied to the elbow. During the preoperative period, we always advise patients to keep on attempting to practice the elbow ROM and forearm rotation by bouncing a ball actively, by taking the affected elbow up and down by themselves, and rolling it forward and backward. A violent training is forbidden while performing a rehabilitation program in case of HO occurrence.

In addition to the retrospective nature of the study, the relatively heterogeneous case series (due to a low prevalence of elbow stiffness) and the fewer number of patients analysed were also limitations, which made more meaningful statistical comparisons among the three groups (especially with respect to complications) difficult. The intra-group (pre- and post-operatively) and inter-group (3 groups post-operatively) evaluations of the elbow muscle and grip-strength were not included in this study, which was also a limitation.

## Conclusion

The results of our retrospective cohort study showed no significant difference during the final follow-up, with respect to various outcomes (such as postoperative elbow motion capacities, functional scores, and post-operative complication rates), among patients who had undergone an early, median, and late open arthrolysis to correct elbow stiffness, following an injury. Therefore, an early arthrolysis is recommended for its multiple advantages. It is advisable to shorten the conventional delay period of > 1 year after the injury.

## Abbreviations

ANOVA: Analysis of variance
CPM: Continuous Passive Motion
HO: Heterotopic ossification
MEPS: Mayo Elbow Performance Score
ROM: Range of motion
VAS: Visual analog scale
